# The Topical Treatment of Tubercular Phthisis

**Published:** 1887-08

**Authors:** 


### *THE TOPICAL TREATMENT OF TUBERCULAR PHTHISIS.*

The researches of Koch and others demonstrate that bacilli are found in larger or smaller numbers in all forms of tuberculosis, and further, that some idea of the extent of the phthisical process may be determined from the number of bacilli. It was found that the bacilli are more abundant where there was recent caseous infiltration, and in the interior of cavities whose walls were undergoing rapid softening. When the walls of the cavities are firm and indurated, the bacilli are few in number, and they are fewest in the cicatricial contracting and pigmented lung-tissue. By examining the sputa of the phthisical patient, much light may be thrown on the condition of the lungs. We may surmise that the fewer the bacilli, the less the destruction, and *vice versa*. If we admit that bacilli are the cause of tuberculosis, bacillicides are naturally indicated. The experience of various physicians of the favorable results obtained from the use of bacillicides at any rate warrants their employment, even though they may not admit that the bacilli are the causation of the disease. Many bacillicides have been recommended; among them is corrosive sublimate, and this Dr. J. L. Porteous (*Edinburgh Medical Journal*, May, 1887,) in his experience has found to be the best.

Various modes have been suggested for applying the remedies; inhalation of the spray containing the bacillicide being the most effectual, as it gets directly to the part affected and retains the original strength of the solution.

The method which Dr. Porteous employs is to use a spray producer with a reservoir graduated to hold the exact quantity to be inhaled at one sitting. The patient is then directed to deeply inspire at the same time that the ball of the vaporizer is squeezed. By this means the vapor is drawn directly into the lungs and is in no way diluted before reaching the part affected. This process is repeated every four or six hours. Dr. Porteous reports three cases in which extremely favorable results followed this method of treatment.—*Therapeutic Gazette.*

